# An Investigation of the Effect of Combining Tolterodine and Duloxetine in the Treatment of Mixed-Type Urinary Incontinence and the Factors Affecting Success

**DOI:** 10.3390/jcm14103575

**Published:** 2025-05-20

**Authors:** Resul Sobay, Eyüp Veli Küçük

**Affiliations:** Department of Urology, Umraniye Training and Research Hospital, İstanbul 34764, Türkiye; eyupveli@gmail.com

**Keywords:** mixed urinary incontinence, duloxetine, tolterodine, combination therapy, urge-predominant subtype

## Abstract

**Background**: Mixed urinary incontinence (MUI), particularly the urge-predominant subtype, involves both stress urinary incontinence (SUI) and urge urinary incontinence (UUI), posing a therapeutic challenge. Duloxetine, a serotonin–norepinephrine reuptake inhibitor (SNRI), enhances urethral tone, while tolterodine, an antimuscarinic agent, reduces detrusor overactivity. Their combination may offer synergistic benefits. **Aim**: The aim of this study was to evaluate the efficacy of duloxetine and tolterodine combination therapy in urge-predominant MUI and identify factors influencing treatment success. **Method**: A retrospective study was conducted on 106 patients (mean age: 56.45 years) with urge-predominant MUI treated with duloxetine (40 mg twice daily) and tolterodine (4 mg once daily) for 12 weeks. Treatment outcomes were evaluated using the overactive bladder symptom score (OABSS), International Consultation on Incontinence Questionnaire Short Form (ICIQ-SF), 24 h pad test, and Clinical Global Impression Scale (CGI). Univariate and multivariate regression analyses were performed to determine predictors of success. **Results**: Significant improvements were observed: OABSS decreased from 11.08 to 6.95, ICIQ-SF decreased from 15.69 to 8.84, and pad use decreased from 3.58 to 0.73/day (all *p* 0.0001). Bladder capacity increased from 315.09 mL to 436.32 mL. Baseline ICIQ-SF scores were independent predictors of success (odds ratio [OR] = 2.919, *p* = 0.001). Patient satisfaction reached 77.4%, with mild side effects (constipation and dizziness) in 14 patients. **Conclusions**: Duloxetine and tolterodine combination therapy significantly improved symptoms and quality of life in urge-predominant MUI. Baseline ICIQ-SF scores may predict treatment success. Further prospective studies are needed.

## 1. Introduction

Mixed urinary incontinence (MUI), characterized by the coexistence of stress urinary incontinence (SUI) and urge urinary incontinence (UUI), presents a significant therapeutic challenge due to its multifaceted etiology [1]. The urge-predominant subtype of MUI is particularly complex, as it involves both involuntary detrusor muscle contractions and compromised urethral sphincter function. This dual pathology often necessitates a combination of therapeutic strategies to effectively manage symptoms and improve quality of life for patients [2].

Duloxetine, a serotonin and norepinephrine reuptake inhibitor, has demonstrated efficacy in enhancing urethral sphincter tone, thereby reducing episodes of SUI [3]. Clinical trials have shown that duloxetine treatment leads to significant reductions in incontinence episode frequency and improvements in quality-of-life measures among women with SUI [4].

Tolterodine, an antimuscarinic agent, is commonly employed to address symptoms of UUI by inhibiting involuntary bladder contractions [5,6]. Studies have indicated that tolterodine extended-release formulations significantly decrease weekly urge incontinence episodes and improve patient perceptions of bladder conditions in women with urge-predominant MUI [7].

Despite advancements in pharmacotherapy and behavioral interventions, the management of MUI remains challenging due to the interplay of stress and urge components. Behavioral therapies, such as pelvic floor muscle training (PFMT), have shown moderate efficacy in improving symptoms, particularly in women with mild-to-moderate MUI [8]. However, adherence to long-term PFMT is often suboptimal, and its benefits may diminish over time without sustained practice [9]. Antimuscarinics like tolterodine and mirabegron (a β3-adrenergic agonist) are effective in reducing UUI episodes but may not sufficiently address SUI, leading to partial symptom relief in MUI patients. Similarly, while duloxetine has demonstrated benefits in SUI by enhancing urethral sphincter activity, its use is limited by side effects such as nausea and dizziness, which contribute to high discontinuation rates [10]. The lack of a single therapeutic agent that effectively targets both SUI and UUI, coupled with limited high-quality evidence on combination therapies, represents a significant gap in MUI management. This study aims to address this gap by evaluating the efficacy of a combined duloxetine and tolterodine regimen, targeting both types of incontinence simultaneously.

Current treatment strategies for MUI often prioritize one incontinence subtype over the other, resulting in incomplete symptom control. A systematic review by Balk et al. (2019) highlighted that combination therapies, including antimuscarinics with duloxetine or PFMT, may offer superior outcomes compared to monotherapy, though high-quality randomized controlled trials remain scarce [11]. Patient-reported outcomes indicate that dissatisfaction with existing treatments often stems from inadequate symptom relief or intolerable side effects, emphasizing the need for personalized approaches.

The rationale for combining duloxetine and tolterodine in treating urge-predominant MUI lies in targeting both components of the condition: duloxetine addresses the stress-related aspect by enhancing urethral closure, while tolterodine mitigates the urge component by reducing detrusor overactivity [12]. Although direct studies on this specific combination are limited, the individual efficacy of each agent suggests potential benefits. Further research is warranted to evaluate the synergistic effects of this combination therapy in managing urge-predominant MUI.

The aim of this study was to investigate the efficacy of tolterodine and duloxetine as a combination treatment in urge-predominant MUI and to determine the factors affecting success.

## 2. Materials and Methods

The data of MUI patients over the age of 18 who applied to the urology clinic (a single-center study) between January 2021 and July 2024 were analyzed retrospectively after obtaining Ethics Committee approval. The study was approved by the ethics committee (Ethic number: 291; 5 September 2024). The study was designed in accordance with the Declaration of Helsinki.

The study strictly enrolled women with urge-predominant MUI, defined as ≥1 stress incontinence episode and ≥3 urge episodes on a 3-day voiding diary, alongside dominant UUI symptoms per patient history. These criteria ensured that all included patients exhibited both SUI and UUI, with urge symptoms being the predominant complaint based on detailed patient interviews and voiding diaries. Exclusion criteria were expanded to include neurological disorders (e.g., Parkinson’s disease and multiple sclerosis) affecting bladder function, uncontrolled diabetes mellitus (HbA1c > 8%), and the concurrent use of medications influencing lower urinary tract function (e.g., diuretics and alpha-blockers).

Patients with active urinary tract infection, urinary tract stones, a history of malignancy in the urinary system, previous surgical treatment for UI, grade 3–4 cystocele, patients with prolapse or a residual urine volume of more than 150 cc were excluded from the study. Patients with only SUI or UUI were also excluded from this study.

Our patients were evaluated with pre-treatment urine analysis, urine culture, ultrasonography (to determine the residual urine volume after urination), and clinical examination (abdominal, pelvic, and perineal exams).

SUI was demonstrated with a provocative stress (cough) test in both lithotomy and standing positions. The type of incontinence was determined with UI questioning, and the dominant symptoms were determined according to the patient’s history. In order to evaluate the degree of symptoms, the overactive bladder symptom score (OABSS) and International Consultation on Incontinence Questionnaire Short Form (ICIQ-SF) were filled in both before and after treatment. Patients were subjected to a 24 h pad test. When necessary, urodynamic evaluation was also performed on these patients. A total of 20 patients with urinary tract infection, urinary tract stones, urinary malignancy, patients with chronic obstructive pulmonary disease, patients who had previously undergone surgical treatment for UI, patients with grade 3–4 cystocele, patients with prolapse, and patients with a residual urine volume of more than 150 cc were excluded from the study, and urodynamic evaluation was performed.

Post-void residual (PVR) urine volume was measured via transabdominal ultrasonography (USG) using a 3.5–5 MHz convex transducer (Mindray DP-50, China) within 10 min of voluntary voiding. Patients were instructed to empty their bladders completely before the measurement, and PVR was calculated using the ellipsoid formula (length × width × height × 0.52). A PVR > 150 mL was an exclusion criterion, as elevated residuals may indicate voiding dysfunction or bladder outlet obstruction, complicating MUI management. To assess urethral hypermobility, a Q-tip test was performed with the patient in the lithotomy position and the bladder filled to 200–400 mL (confirmed via USG). A sterile, lubricated cotton swab was inserted into the urethra to the level of the bladder neck, and the resting angle relative to the horizontal was recorded. During maximal Valsalva or cough, the change in angle (≥30° indicated significant hypermobility) was documented, correlating with intrinsic sphincter deficiency in SUI.

Patients were given tolterodine at 4 mg 1×1 (Detrusitol, Viatris Specialty LLC, Canonsburg, PA, USA) and duloxetine at 40 mg 2×1 (Nexetin 40 mg, Nobel İlaç, Istanbul, Türkiye). The dosage of tolterodine (4 mg once daily) was selected based on its established efficacy and tolerability in reducing UUI episodes, as demonstrated in prior studies [6]. Duloxetine (40 mg twice daily) was chosen to maximize urethral sphincter enhancement while balancing side effect risks, consistent with clinical guidelines for SUI management [3]. Treatment responses of the patients were evaluated after 12 weeks. Side effects were recorded. Patient satisfaction with the treatment was evaluated with the Clinical Global Impression Scale (CGI).

The OABSS is a questionnaire consisting of 4 questions that evaluate overactive bladders and urge urinary incontinence [13]. The questionnaire can be scored between 0 and 15 points. A minimum score of 3 points and 2 points from Question 3 confirms the diagnosis of an overactive bladder. As the score from the questionnaire increases, the severity of UUI also increases. Linguistic validation was performed by Culha et al. [14].

ICIQ-SF is a questionnaire form consisting of 3 questions related to urinary incontinence. The questionnaire can be scored between 0 and 21, and the higher the score, the higher the severity of urinary incontinence. Language validation was performed by Çetinel et al. [15].

For the pad test, patients were weighed and given dry pads. Twenty-four hours later, the patients’ pads were weighed again, and the difference was recorded as the amount of urine missed per day.

### Statistical Analysis

Data analysis was performed using the SPSS 25.0 (IBM, Armonk, NY, USA) program. The distribution of data was evaluated using the Kolmogorov–Smirnov test. For pre- and post-treatment evaluation, a dependent variable *t*-test and Fisher’s exact test were used. Univariate and multivariate analyses were performed for factor analysis. A significant *p*-value was determined as <0.05.

## 3. Results

This study included 106 participants with a mean age of 56.45 years (SD = 9.08, range: 42–72). The mean BMI (Body Mass Index) was 26.34 (SD = 3.02), and the average parity was 2.55 (SD = 1.17). The duration of symptoms prior to treatment was 27.68 months (SD = 10.29), with baseline OABSS and ICIQ-SF scores of 11.08 (SD = 1.61) and 15.69 (SD = 2.52), respectively. All patients exhibited both SUI (≥1 episode) and UUI (≥3 episodes), with urge symptoms being predominant per the inclusion criteria. These demographic and clinical characteristics are summarized in Table 1.

Significant improvements were observed in all measured outcomes following treatment (Table 2). The OABSS decreased from 11.08 ± 1.61 to 6.95 ± 1.55 (*p* = 0.0001), and the ICIQ-SF score improved from 15.69 ± 2.52 to 8.84 ± 2.67 (*p* = 0.0001). Symptom frequency and severity also showed marked reductions, including pad use per day (3.58 ± 1.07 to 0.73 ± 1.05, *p* = 0.0001), micturition episodes (11.46 ± 2.21 to 7.44 ± 1.84, *p* = 0.0001), and nocturia (2.59 ± 1.01 to 0.61 ± 0.76, *p* = 0.0001). Additionally, mean bladder volume increased significantly from 315.09 ± 64.43 mL to 436.32 ± 97.23 mL (*p* = 0.0001), indicating improved bladder capacity post-treatment.

Univariate analysis (Table 3) identified several factors associated with treatment outcomes, including parity (OR = 0.985, *p* = 0.049), the ICIQ-SF score (OR = 3.812, *p* = 0.005), incontinence episodes (OR = 1.550, *p* = 0.037), and pad use per day (OR = 1.071, *p* = 0.008). However, in the multivariate analysis, only the ICIQ-SF score remained a significant predictor (OR = 2.919, 95% CI: 1.707–4.994, *p* = 0.001), suggesting its independent association with treatment efficacy. Other variables, such as age, BMI, and comorbidities (DM, HT, CAD), showed significant associations in either analysis.

When the patients’ satisfaction was questioned according to the CGI scale, 82 of the patients (77.40%) stated partial or complete recovery. No patient was excluded from the study during the 12 weeks. Side effects were observed in 14 patients. The most common of these effects were constipation in eight patients, dizziness and nausea in four patients, and dry mouth in two patients. No patient discontinued treatment due to side effects.

## 4. Discussion

The findings of this study demonstrate significant improvements in symptoms and quality of life among patients with urge-predominant mixed urinary incontinence (MUI) following combination therapy with duloxetine and tolterodine. The reductions in OABSS and ICIQ-SF scores, along with decreased pad use, micturition frequency, and nocturia, underscore the efficacy of this dual pharmacological approach. Notably, the multivariate analysis identified the ICIQ-SF score as an independent predictor of treatment success, suggesting that baseline symptom severity may influence therapeutic outcomes. These results align with previous research, highlighting the benefits of targeting both the stress and urge incontinence components in MUI [16]. Other treatment modalities, such as sacral nerve stimulation, have also shown promise in MUI management by modulating neural pathways to improve bladder control [17]. However, these interventions are invasive and may not be suitable for all patients, highlighting the advantage of pharmacological combination therapies like duloxetine and tolterodine for broader applicability.

The therapeutic rationale for combining duloxetine and tolterodine lies in their complementary mechanisms of action. Duloxetine, a serotonin–norepinephrine reuptake inhibitor (SNRI), enhances urethral sphincter tone by increasing central noradrenergic activity, thereby improving stress-related leakage [3]. Tolterodine, an antimuscarinic agent, reduces detrusor overactivity by blocking muscarinic receptors in the bladder, addressing the urge component [18]. This dual-pathway approach is particularly relevant for urge-predominant MUI, where both mechanisms contribute to symptom persistence. The observed improvements in bladder volume further support the hypothesis that combined therapy may restore normal bladder function more effectively than monotherapy.

The synergistic effects of duloxetine and tolterodine may explain the pronounced symptom relief seen in this study. While duloxetine alone has shown efficacy in stress incontinence and tolterodine in urge incontinence, its combination with tolterodine appears to address the complex pathophysiology of MUI more comprehensively. Previous studies on monotherapies have reported moderate improvements, but the current results suggest superior outcomes with dual therapy, particularly in reducing incontinence episodes and nocturia [19,20]. The high patient satisfaction rate (77.4%) and low discontinuation rate due to side effects further reinforce the clinical viability of this approach.

When compared to the existing literature, the current findings are consistent with studies reporting the benefits of antimuscarinics and SNRIs in MUI, but with notable enhancements. For instance, a trial by Kreder et al. demonstrated that tolterodine alone reduces urge incontinence episodes by 50%, whereas the present study achieved a 79% reduction in pad use, suggesting additive benefits from duloxetine [7]. Similarly, the improvement in ICIQ-SF scores surpassed those reported in studies using single-agent therapy, reinforcing the value of combination treatment [16]. These comparisons highlight the potential for dual therapy to bridge gaps in MUI management where monotherapy falls short.

The accompanying study highlights the significant benefits of combining duloxetine and tolterodine for urge-predominant MUI, demonstrating marked improvements in symptom severity, quality of life, and bladder capacity. By simultaneously targeting stress (via duloxetine’s urethral tone enhancement) and urge (via tolterodine’s detrusor suppression) components, this dual-therapy approach addresses the multifactorial pathophysiology of MUI more comprehensively than monotherapies. The study’s findings—reduced pad use, lower OABSS/ICIQ-SF scores, and high patient satisfaction (77.4%)—suggest that such combinations may bridge the gap in MUI management, where single-agent therapies often yield incomplete relief. Notably, the baseline ICIQ-SF score emerged as a predictor of success, underscoring the value of personalized treatment stratification.

Despite these promising results, this study has several limitations that warrant consideration. The retrospective design and absence of a control or comparator group (e.g., monotherapy or placebo) limit causal inferences, as improvements could partially result from natural symptom variation or placebo effects. Additionally, the exclusion of patients with severe comorbidities prior to surgical interventions or high-grade prolapse restricts the generalizability of the findings to a broader MUI population, particularly those with complex clinical profiles encountered in real-world practice. The lack of a validated comorbidity index (e.g., Charlson Comorbidity Index) in the analysis further limits the ability to account for potential confounders, as only select comorbidities (diabetes, hypertension, and coronary artery disease) were assessed. The relatively short follow-up period of 12 weeks precludes the assessment of long-term efficacy and side effect profiles. Future randomized controlled trials with extended follow-up, control groups, and comprehensive comorbidity assessments are needed to validate these findings and explore durability. Comparative studies involving other combination therapies (e.g., mirabegron with duloxetine) could further clarify optimal treatment strategies [16].

Looking ahead, future MUI treatment strategies should prioritize long-term efficacy studies and comparative trials assessing emerging therapies (e.g., mirabegron with duloxetine or novel β3-agonists). Advances in precision medicine, such as biomarkers for subtype classification, could refine patient selection for combination therapies. Additionally, integrating non-pharmacological interventions (e.g., neuromodulation or personalized pelvic floor therapy) may further optimize outcomes.

Despite these promising results, this study has limitations. The retrospective design and lack of a control group limit causal inferences, and the relatively short follow-up (12 weeks) precludes the assessment of long-term efficacy and side effects. Additionally, the exclusion of patients with severe comorbidities or prior surgeries may limit generalizability. Future randomized controlled trials with extended follow-up are needed to validate these findings and explore durability. Furthermore, comparative studies involving other combination therapies (e.g., mirabegron with duloxetine) could clarify optimal treatment strategies [16].

## 5. Conclusions

In conclusion, the combination of duloxetine and tolterodine significantly improves symptoms and quality of life in patients with urge-predominant MUI, with the ICIQ-SF score emerging as a key predictor of success. The dual mechanism of action addresses both stress and urge components, offering a more comprehensive solution than monotherapy. Future research should focus on long-term outcomes and head-to-head comparisons with emerging therapies to refine clinical guidelines for MUI management.

## Figures and Tables

**Table 1:** Characteristics.

N = 106	Mean	SD	Min	Max
Age (years)	56.45	9.08	42	72
BMI (kg/m^2^)	26.34	3.02	19.83	33.67
Parity (*n*)	2.55	1.17	0	5
Duration (months)	27.68	10.29	6	48
OABSS (score)	11.08	1.61	8	15
ICIQ-SF (score)	15.69	2.52	11	21

**Table 2:** Comparison between pre-treatment and post-treatment scores.

	Before Treatment	After Treatment	*p*
OABSS	11.08 ± 1.61	6.95 ± 1.55	0.0001
ICIQ-SF	15.69 ± 2.52	8.84 ± 2.67	0.0001
Pad/day	3.58 ± 1.07	0.73 ± 1.05	0.0001
Micturition/day	11.46 ± 2.21	7.44 ± 1.84	0.0001
Frequency/day	15.26 ± 2.86	8.76 ± 2.64	0.0001
Incontinence/day	5.18 ± 1.45	0.93 ± 1.39	0.0001
Nocturia/day	2.59 ± 1.01	0.61 ± 0.76	0.0001
Mean bladder volume	315.09 ± 64.43	436.32 ± 97.23	0.0001

**Table 3:** Univariate and multivariate analysis.

	Univariate Analysis			Multivariate Analysis		
	OR	CI	*p*	OR	CI	*p*
Age	1.380	0.953–2.000	0.088			
BMI	0.460	0.167–1.218	0.134			
Parity	0.985	0.035–0.989	0.049	0.758	0.422–1.359	0.352
DM	0.033	0.364–3.684	0.156			
HT	1.121	0.876–1.344	0.125			
CAD	3.091	1.112–5.475	0.082			
Duration	1.903	0.509–7.113	0.339			
OABSS	0.701	0.079–6.244	0.751			
ICIQ-SF	3.812	0.973–5.810	0.005	2.919	1.707–4.994	0.001
Frequency	2.391	0.565–10.120	0.236			
Incontinence	1.550	0.243–3.231	0.037	1.237	0.514–2.979	0.606
Nocturia	0.098	0.004–2.393	0.154			
Pad/day	1.071	0.235–1.388	0.008	0.499	0.145–1.718	0.271

## Data Availability

The datasets analyzed during the current study are available from the corresponding author upon reasonable request.

## Abbreviations

The following abbreviations are used in this manuscript:

MUI	Mixed urinary incontinence
SUI	Stress urinary incontinence
UUI	Urge urinary incontinence
OABSS	Overactive bladder symptom score
ICIQ-SF	International Consultation on Incontinence Questionnaire Short Form
CGI	Clinical Global Impression Scale
BMI	Body Mass Index

## References

[1] Rogers R.G., Pauls R.N., Thakar R., Morin M., Kuhn A., Petri E., Fatton B., Whitmore K., Kinsberg S., Lee J. (2018). An International Urogynecological Association (IUGA)/International Continence Society (ICS) joint report on the terminology for the assessment of sexual health of women with pelvic floor dysfunction. Neurourol. Urodyn..

[2] Minassian V.A., Devore E., Hagan K., Grodstein F. (2013). Severity of urinary incontinence and effect on quality of life in women by incontinence type. Obstet. Gynecol..

[3] Mariappan P., Alhasso A., Ballantyne Z., Grant A., N’Dow J. (2007). Duloxetine, a serotonin and noradrenaline reuptake inhibitor (SNRI) for the treatment of stress urinary incontinence: A systematic review. Eur. Urol..

[4] Bent A.E., Gousse A.E., Hendrix S.L., Klutke C.G., Monga A.K., Yuen C.K., Muram D., Yalcin I., Bump R.C. (2008). Duloxetine compared with placebo for the treatment of women with mixed urinary incontinence. Neurourol. Urodyn..

[5] Khullar V., Amarenco G., Angulo J.C., Cambronero J., Høye K., Milsom I., Radziszewski P., Rechberger T., Boerrigter P., Drogendijk T. (2013). Efficacy and tolerability of mirabegron, a β(3)-adrenoceptor agonist, in patients with overactive bladder: Results from a randomised European-Australian phase 3 trial. Eur. Urol..

[6] Khullar V., Hill S., Laval K.-U., Schiøtz H.A., Jonas U., Versi E. (2004). Treatment of urge-predominant mixed urinary incontinence with tolterodine extended release: A randomized, placebo-controlled trial. Urology.

[7] Kreder K.J., Brubaker L., Mainprize T. (2003). Tolterodine is equally effective in patients with mixed incontinence and those with urge incontinence alone. BJU Int..

[8] Dumoulin C., Cacciari L.P., Hay-Smith E.J.C. (2018). Pelvic floor muscle training versus no treatment, or inactive control treatments, for urinary incontinence in women. Cochrane Database Syst. Rev..

[9] Alewijnse D., Metsemakers J.F.M., Mesters I.E.P.E., van den Borne B. (2003). Effectiveness of pelvic floor muscle exercise therapy supplemented with a health education program to promote long-term adherence among women with urinary incontinence. Neurourol. Urodyn..

[10] Di Nicola M., Dell B., Peduto I., Cipelli R., Pugliese A.C., Signorelli M.S., Ventriglio A., Martinotti G. (2023). Adherence to, and Persistence of, Antidepressant Therapy in Patients with Major Depressive Disorder: Results from a Population-based Study in Italy. Curr. Neuropharmacol..

[11] Balk E.M., Rofeberg V.N., Adam G.P., Kimmel H.J., Trikalinos T.A., Jeppson P.C. (2019). Pharmacologic and Nonpharmacologic Treatments for Urinary Incontinence in Women: A Systematic Review and Network Meta-analysis of Clinical Outcomes. Ann. Intern. Med..

[12] Nygaard I.E. (2019). Evidence-Based Treatment for Mixed Urinary Incontinence. JAMA.

[13] Homma Y., Yoshida M., Seki N., Yokoyama O., Kakizaki H., Gotoh M., Yamanishi T., Yamaguchi O., Takeda M., Nishizawa O. (2006). Symptom assessment tool for overactive bladder syndrome–Overactive bladder symptom score. Urology.

[14] Culha M.G., Degirmentepe R.B., Ozbir S., Cakir S.S., Homma Y. (2019). Turkish validation of the overactive bladder symptom score (OABSS) and evaluation of mirabegron treatment response. Int. Urogynecol. J..

[15] ÇEtİNel B., ÖZkan B., Can G. (2004). ICIQ-SF Türkçe versiyonu validasyon (geçerlilik) çalışması. Türk Urol. Derg./Turk. J. Urol..

[16] Ariman A., Merder E., Çulha M.G. (2021). The effect of Mirabegron and Duloxetine combination in mixed-type urinary incontinence treatment. Rev. Assoc. Med. Bras. (1992).

[17] Siegel S., Noblett K., Mangel J., Griebling T.L., Sutherland S.E., Bird E.T., Comiter C., Culkin D., Bennett J., Zylstra S. (2017). Sacral nerve stimulation for urinary incontinence: A review of clinical effectiveness and guidelines. Neurourol. Urodyn..

[18] Chapple C.R., Van Kerrebroeck P.E., Jünemann K.-P., Wang J.T., Brodsky M. (2008). Comparison of fesoterodine and tolterodine in patients with overactive bladder. BJU Int..

[19] Game X., Dmochowski R., Robinson D. (2023). Mixed urinary incontinence: Are there effective treatments?. Neurourol. Urodyn..

[20] Dmochowski R.R., Sand P.K., Zinner N.R., Gittelman M.C., Davila G.W., Sanders S.W., Transdermal Oxybutynin Study Group (2003). Comparative efficacy and safety of transdermal oxybutynin and oral tolterodine versus placebo in previously treated patients with urge and mixed urinary incontinence. Urology.

